# Confirming an antiphasic bicyclic pattern of forward entrainment in signal detection: A reanalysis of Sun et al. (2021)

**DOI:** 10.1111/ejn.15816

**Published:** 2022-09-14

**Authors:** Kourosh Saberi, Gregory Hickok

**Affiliations:** ^1^ Department of Cognitive Sciences University of California, Irvine Irvine California USA; ^2^ Department of Language Science University of California, Irvine Irvine California USA

**Keywords:** attention, periodicity, phase, psychophysics, signal detection

## Abstract

Forward entrainment refers to that part of the entrainment process that persists after termination of an entraining stimulus. Hickok et al. (2015) reported forward entrainment in signal detection that lasted for two post‐stimulus cycles. In a recent paper, Sun et al. (2021) reported new data which suggested an absence of entrainment effects (Eur. J. Neurosci, 1–18, doi.org/10.1111/ejn.15367). Here we show that when Sun et al.'s data are analysed using unbiased detection‐theoretic measures, a clear antiphasic bicyclic pattern of entrainment is observed. We further show that the measure of entrainment strength used by Sun et al., the normalized Fourier transform of performance curves, is not only erroneously calculated but is also unreliable in estimating entrainment strength due to signal‐processing artifacts.

## Graphical abstract

Forward entrainment refers to that part of the entrainment process that persists after termination of an entraining stimulus. In a recent paper, Sun et al. (2021) reported new data which suggested an absence of entrainment effects (Eur. J. Neurosci, 1–18, doi.org/10.1111/ejn.15367). Here, we show that when Sun et al.'s data are analysed using unbiased detection‐theoretic measures, a clear antiphasic bicyclic pattern of entrainment is observed.

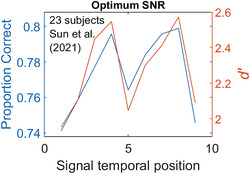

**Abbreviations**
AM: amplitude modulation
FA: false alarm
FFT: fast Fourier transform
FM: frequency modulation
SKE: signal‐known‐exactly
SNR: signal‐to‐noise ratio

## 1 INTRODUCTION

In an auditory signal‐detection study, Hickok et al. (2015) showed that a sinusoidally amplitude‐modulated noise can entrain the detection of a signal in steady‐state noise presented after termination of the entraining stimulus. We use the term ‘forward entrainment’ to refer to this and other similar phenomena in which the entrainment process outlasts the entraining stimulus (Saberi & Hickok, 2021, 2022). Sun et al. (2021) recently reported the results of experiments in which they used stimuli identical to those of Hickok et al. to investigate forward entrainment in signal detection. Their main finding was that while there was an inverse U‐shape pattern in post‐stimulus performance, no bicyclic (M‐shaped) pattern was observed at the overall group level, although approximately a third of their subjects did show forward entrainment but with variable phase alignment across subjects. In what follows, we describe some of the methodological differences as well as data‐analysis dissimilarities between Hickok et al. and Sun et al. which may help in understanding their discrepant findings.

## 2 A DETECTION THEORY ANALYSIS

One of the main experimental design features in Hickok et al. (2015) was the introduction of trial‐by‐trial level uncertainty by randomly selecting the signal level from one of five intensities. In their first experiment, Sun et al. used only two signal levels. This is important because Farahbod et al. (2020) have shown that level uncertainty appears to be critical to observing forward entrainment, at least in the signal‐detection design used by Hickok et al. (2015). Because of this difference in design (among other methodological differences), Sun et al. referred to their first experiment as a ‘conceptual replication’. Based on Farahbod et al.'s (2020) finding on uncertainty, Sun et al. then ran a second experiment which they referred to as an ‘exact’ replication. However, even in this case, there were nearly a half dozen methodological, procedural and experimental design differences between their exact replication and the original Hickok et al. (2015) study. Some of these differences are outlined in the footnotes section. 1

The most critical difference between the two studies, however, is in their data‐analysis approach. In the main part of their manuscript, Sun et al. used ‘hit rates’ as a dependent measure, restricting their analysis only to ‘signal trials’ (trials that contained a tone to be detected) and eliminating from analysis all no‐signal trials. By excluding no‐signal trials, Sun et al. effectively used half as many trials as Hickok et al. who evaluated performance using ‘proportion correct’ as the dependent measure. This latter measure includes both signal and no‐signal trials. Sun et al.'s reasoning for excluding no‐signal trials was to reduce ‘random response noise’. What they refer to as ‘random noise’, however, is in reality a measure of false‐alarm (FA) rate which is just as informative as hit rate in evaluating performance. Exclusive reliance on hit rates in a *single‐interval yes–no task* is a critical flaw that produces inaccurate results unless FAs are also taken into account. In an extreme case, a subject could simply respond ‘yes’ on every trial and get 100% hit rate without attending to the stimulus. Use of ‘hit rate’ as a dependent measure by itself is therefore both misleading and biased. In fact, signal detection theory emerged originally in engineering and then extended to psychophysics in reaction to flaws inherent in measurements exclusively based on hit rates (Green & Swets, 1966; Tanner & Birdsall, 1958).

**FIGURE 1:**
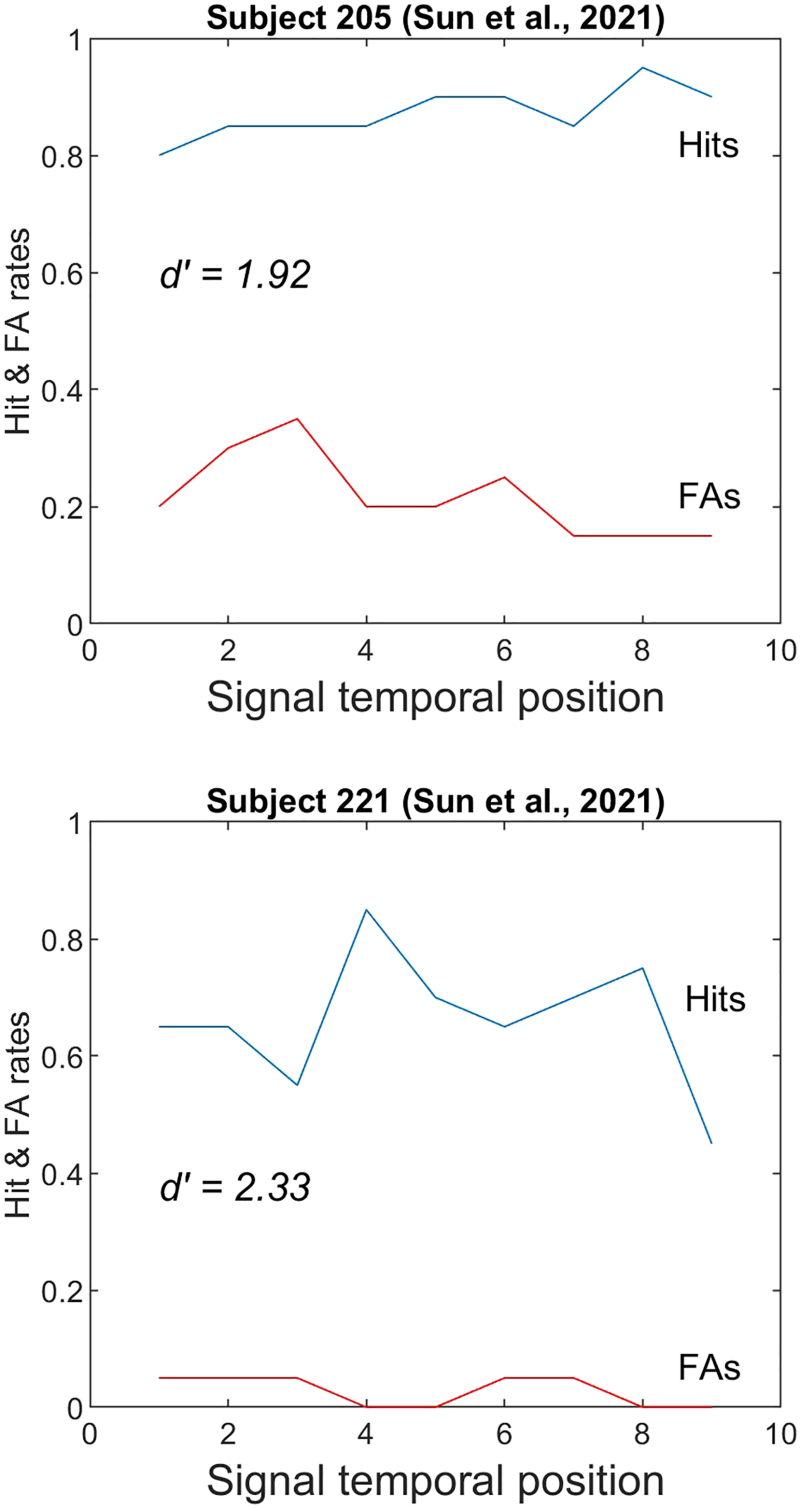
Hit and false‐alarm rates for two subjects from Sun et al. (2021). Subject 205 (top) has a higher averaged hit rate but lower *d*′ than subject 221 (bottom) when false alarms are taken into account in calculating an unbiased measure of performance.

Figure 1 shows hit‐rate data from Sun et al. (2021) plotted individually for two subjects from their experiment 2. Also included are their FA rates (not shown in Sun et al.'s original paper). A cursory examination illustrates our concern. Subject 205 has an average hit rate of 0.87, and subject 221 has an average hit rate of 0.66. If we use an unbiased measure of performance, such as the detection index *d′*, it becomes clear that subject 221 is actually significantly outperforming subject 205 in detecting the tonal signal (*d′* = 2.33 vs. 1.92). The problem of course is that subject 205 has a liberal decision criterion that results in a disproportionately large number of ‘yes’ responses (hence the high FA rates for this subject), whereas subject 221 is much more strict (or careful) in reporting ‘yes’, as evident from this subject's very low FA rates. This problem is also observed in the data of several other subjects from Sun et al., for example subjects 202, 217 and 219 all have significantly lower hit rates, but higher performance levels than subject 205 when evaluating performance using an unbiased measure (*d′*).

### 2.1 Nonlinear relationship between *d′* and hit rate

Sun et al. (2021) state that FAs serve only to add noise to measurements and imply that, if taken into account, function as a scalar for performance. They consequently claim that incorporating FAs into analysis will not affect the *shape* of the *d′* curves relative to hit‐rate curves. There is, however, a statistical nonlinearity between *d′* and hit (or FA) rates that alters performance curves in precisely the direction that would diminish detection of bicyclic patterns if based alone on hit rates instead of *d′* (i.e., which takes into account both hit and FA rates). This nonlinear relationship may be expressed as the quantile function:

(1) $$d^{\prime }=\Phi^{-1}\left[P\left(Y|\mathrm{SN}\right)\right]-\Phi^{-1}\left[P\left(Y|N\right)\right]\;P\in \left(0,1\right)$$

where $\Phi^{-1}$ is the inverse cumulative Gaussian distribution, $P\left(Y|\mathrm{SN}\right)$ is the probability of responding ‘signal’ (or ‘yes’) on signal‐plus‐noise trials (hit rate) and $P\left(Y|N\right)$ is the probability of responding ‘signal’ on noise‐alone trials (FA rate). The subject's *decision* is in fact based not on characteristics of the signal‐plus‐noise trials alone (as Sun et al. assume) but on the likelihood ratio:

(2) $$l\left(x\right)=e^{-0.5{\left(\frac{x-\mu_{s}}{\sigma }\right)}^{2}}/e^{-0.5{\left(x/\sigma \right)}^{2}}$$

which is the ratio of the height of the probability density function under the signal‐plus‐noise distribution (with expected value $\mu_{s}$) for a given sensory observation (*x*) relative to that height for the noise‐alone distribution. To maximize detection performance, the observer's decision rule in a single‐interval yes–no signal‐detection task is

(3) $$D=Y\;\mathrm{iff}\;l\left(x\right)\geq \beta$$

If the likelihood ratio is equal or greater than the decision criterion ( β), the subject responds ‘yes’ (there was a ‘signal’); otherwise they respond that the observation was obtained from the noise‐alone distribution and will respond ‘no’. The position of the decision criterion β determines the degree to which the observer is biased in their decision. Sun et al. incorrectly assume that subjects base their decisions on the probability density associated with the signal‐plus‐noise distribution instead of the likelihood ratio and the cumulative evidence across trials under both distributions.

**FIGURE 2:**
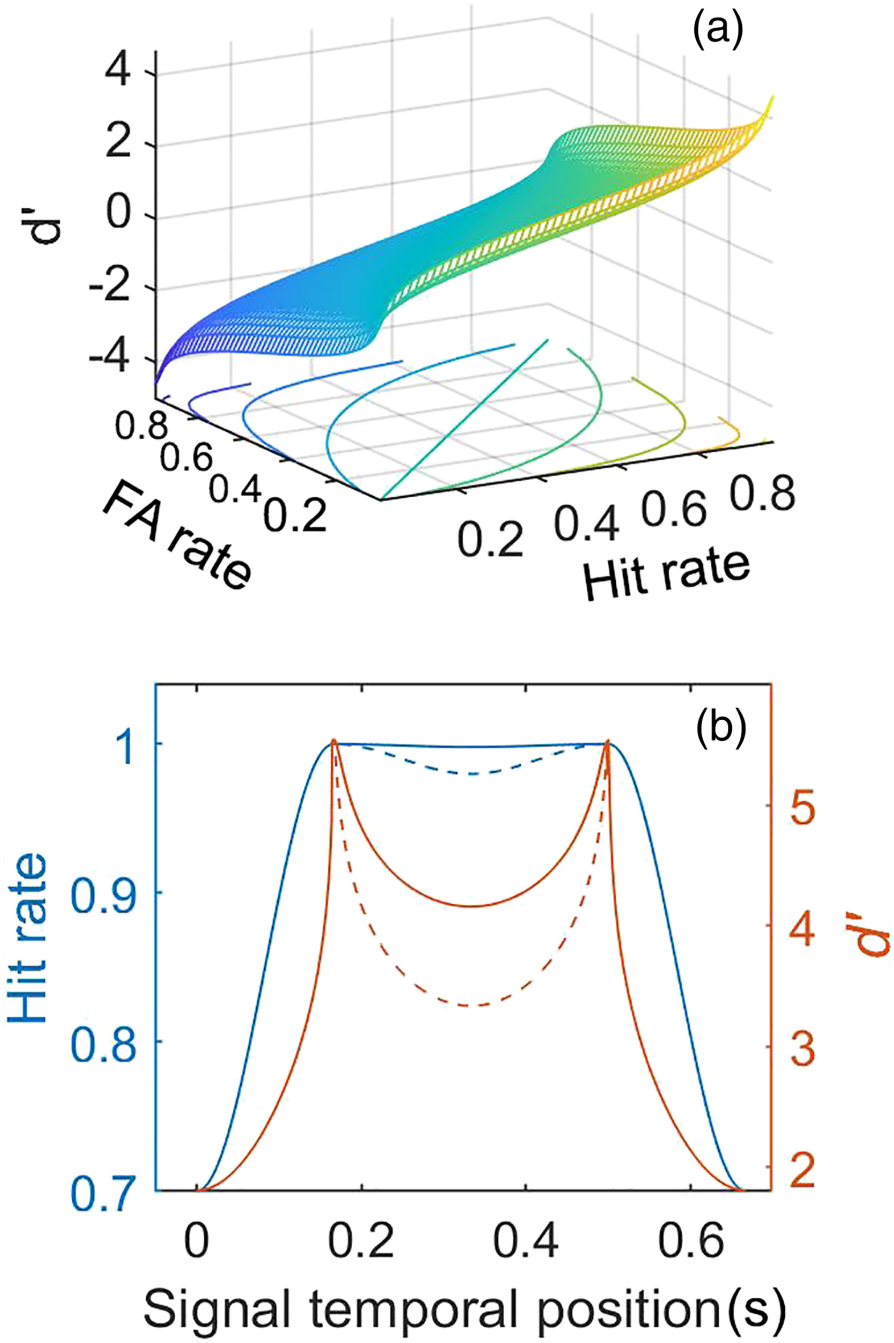
(a) Nonlinear relation between *d*′ and hit and FA rates. (b) Small changes in hit rate may result in large changes in *d*′ (see text).

The nonlinear transform of *d′* described in Equation 1 is plotted in Figure 2a as a joint function of hit and FA rates. Figure 2b shows that small (even imperceptible) perturbation in hit rate can translate into significant modulatory effects in *d′* space (FA rate = 0.1). Although we have shown extreme cases here, the point raised is simply that use of hits rates without accounting for FAs (even a constant FA rate) will, among other problems, reduce the likelihood of detecting modulatory patterns in performance. Furthermore, *averaging* hit‐rate curves across subjects, as Sun et al. have done, is misleading because the averaging process applies a linear operant to nonlinear space. A hit rate of 0.9 compared to 0.8 does not represent the same difference in sensitivity as a hit rate of 0.8 relative to 0.7, whereas differences in *d′* are normalized across the entire range of detection curves. The plots shown in Figure 2b are for a fixed value of FA (0.1). Different values of FA give rise to a family of nonlinear curves that define the relationship between *d′* and hit rates.

## 3 REANALYSIS OF SUN ET AL.'S DATA

**FIGURE 3:**
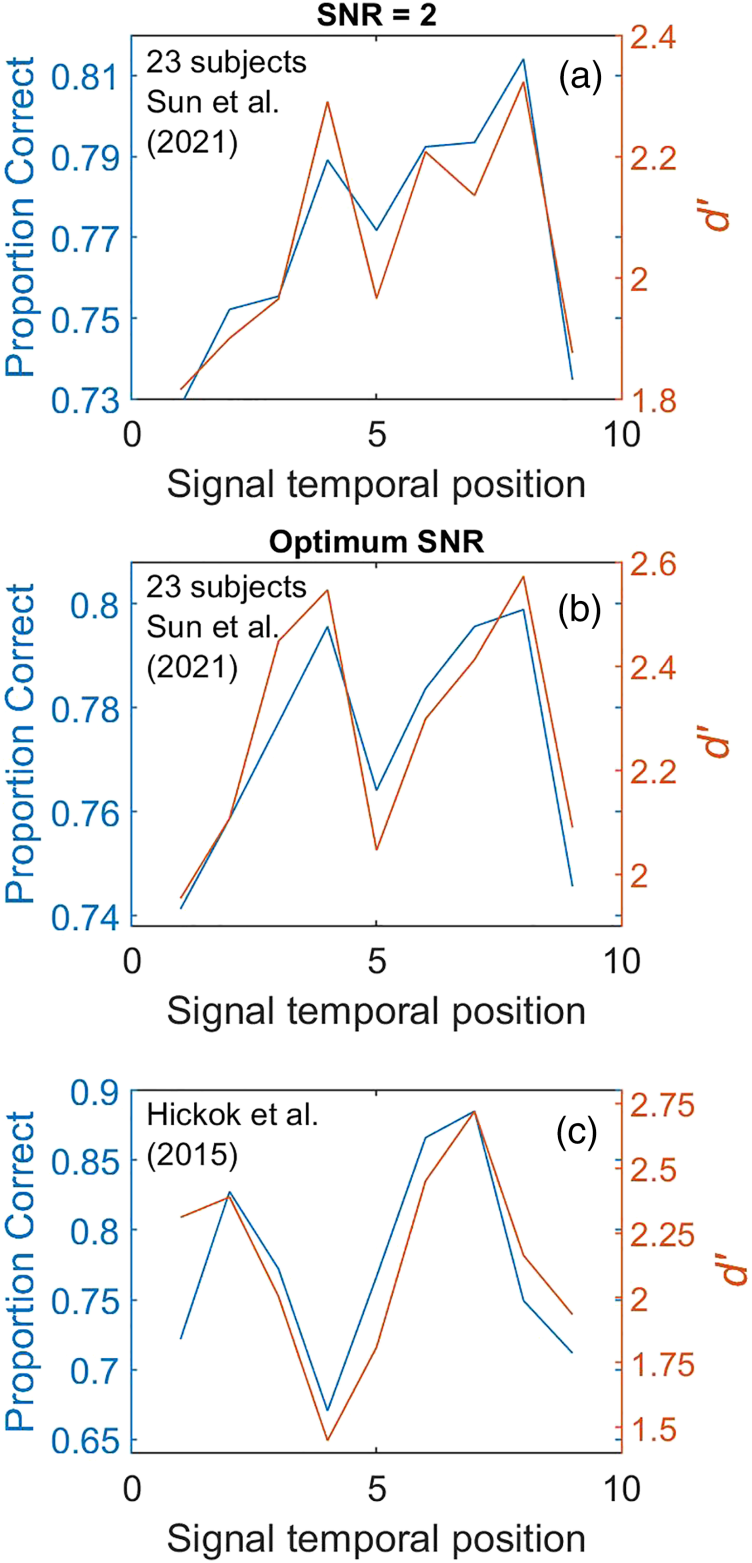
(a) Data from Sun et al. (2021) plotted as proportion correct and as an unbiased index of detectability (*d*′) instead of hit rates averaged across 23 subjects. Note the antiphasic dip at temporal position 5. (b) *d*′ and proportion correct for the same 23 subjects at optimum SNR. (c) Data from Hickok et al. (2015)

To assess the effects of using a biased versus unbiased measure on their results, we reanalysed the raw data of Sun et al. (2021) using a detection‐theoretic approach. For each of 23 subjects, we measured *d′* and proportion correct at each temporal position. 2 We used the same signal level as that used for analysis by Sun et al. (2021) and Hickok et al. (2015), i.e., signal‐to‐noise ratio (SNR) labeled 2 (3 dB). The averaged curve for the 23 subjects is shown in Figure 3a as detection index *d′* and as proportion correct. 3 A reasonably bicyclic (M‐shaped) pattern of performance is observed when an unbiased measure *d′* is used instead of ‘hit rates’, with a dip at temporal position 5 which is precisely antiphasic to the expected modulation peak. A similar, though less prominent, M‐shaped pattern is observed for proportion correct.

A repeated‐measures analysis of variance (ANOVA) on the individual‐subject *d′*s of Figure 3a shows a highly significant effect of temporal position (*F*(8, 176) = 2.85, *p* = 0.005; *η* ^2^ = 0.12). Post hoc paired‐samples *t* tests (one‐tailed) showed significant differences between temporal positions 5 (dip) and 8 (right peak) (*t*(22) = 2.31, *p* = 0.015), a near‐significant difference between temporal positions 4 (left peak) and 5 (dip) (*t*(22) = 1.69, *p* = 0.054), and as expected, no significant difference between the two peaks at temporal positions 4 and 8 (*t*(22) = 0.21, *p* = 0.42). There was no significant difference between the three dips at positions 1, 5 and 9 (*F*(2, 44) = 0.39, *p* = 0.682). Omitting just a *single* outlier subject from analysis and repeating the same statistical analyses on the remaining 22 subjects confirmed a bicyclic pattern with a significant main effect of temporal position (*F*(8, 168) = 3.35, *p* = 0.001), significant difference between temporal positions 4 (left peak) and 5 (dip) (*t*(21) = 2.41, *p* = 0.013), significant difference between positions 5 (dip) and 8 (right peak) (*t*(21) = 2.95, *p* = 0.004) and no significant difference between the two peaks at positions 4 and 8 (*t*(21) = 0.031, *p* = 0.49). There was also no significant difference between the three dips at positions 1, 5 and 9 when analysing the data of these 22 subjects (*F*(2, 42) = 0.23, *p* = 0.796). 4

In light of the large intersubject variability reported by Sun et al., with some subjects averaging very low hit rates (e.g., 25% for subject 218) and others very high (87% for subject 205) at the *same* SNR, we wondered whether some subjects may have performed more optimally at a different SNR. Sun et al. measured performance at five SNRs (spaced ~3 dB apart) but reported results only for a single SNR. We therefore conducted additional analyses on Sun et al.'s data in which we selected for each of 23 subjects the SNR that had generated the strongest modulatory pattern. This approach proved to be quite informative. Figure 3b shows that optimum SNR analysis produces a deeper bicyclic pattern with a statistically significant main effect (*d′* curves) (*F*(8, 176) = 4.27, *p* < 0.001), a highly significant difference between the left peak and antiphasic dip (*t*(22) = 2.87, *p* = 0.004), a highly significant difference between the right peak and antiphasic dip (*t*(22) = 3.80, *p* < 0.001) and no significant difference between the two peaks (*t*(22) = 0.167, *p* = 0.43). 5 For comparison, the data of Hickok et al. (2015) are plotted in Figure 3c as both *d′* and proportion correct.

### 3.1 FA rates and signal temporal uncertainty

There is long‐standing precedent in the literature for calculating *d′* for detection of probe signals randomly positioned in long‐duration maskers (Bonino et al., 2013; Egan et al., 1961; Leibold & Buss, 2020; Lowe & Earle, 1969; Sorkin, 1965; Werner et al., 2009). Egan et al. (1961), for example, in an influential paper on temporal uncertainty, estimated *d′*s from hit and FA rates in a single‐interval signal‐detection task for a 0.5 s tone presented at random (uncertain) times in an 8 s masking noise. FA rates were calculated from ‘yes’ responses to the full 8 s no‐signal trials. Sorkin (1965) measured *d′* for signals with stimulus parameters nearly identical to ours (and Sun et al.'s), that is, a 50 ms 1 kHz tone signal presented at random times within a 600 ms noise window in a single‐interval yes–no task. FAs were calculated form ‘yes’ responses during the 600 ms no‐signal trials. Similarly, Lowe and Earle (1969) measured *d′* in a single‐interval yes–no task from hit and FA rates for an 85 ms 1 kHz tone embedded at uncertain times within a 4 s noise masker. More recently, Werner et al. (2009) reported *d′*s estimated from hit and FA rates for a 300 ms 1 kHz tone positioned at random times in 4 s masking noise, Bonino et al. (2013) measured *d′* for a 120 ms 1 kHz tone occurring at uncertain times in a 600 ms masking interval and Leibold and Buss (2020) reported *d′*s for a 1 s 1 kHz frequency modulated (FM) tone presented at random times in a 4 s speech masking stream. What all these studies (and many others) have in common is that *d′* was estimated for brief tone pulses using FA rates calculated from maskers that were up to 20 times the duration of the signal.

What, however, is the consequence of calculating FA rates based on ‘yes’ responses drawn randomly (and independently) from ‘no‐signal’ trials and assigned to a given temporal position *if* there is reason to believe that FA rates are not uniform throughout the duration of the extended noise masker? How will this affect the current analysis? One of two cases must be true about the experimental paradigm used by Hickok et al., 2015 (and Sun et al.): (1) There is no modulatory effect of the entraining stimulus (i.e., the null hypothesis stands) yielding a uniform FA rate across all nine temporal positions (no matter which part of the long‐duration noise a subject attends to during the noise‐alone trials). That is, if we were to somehow sort out those *noise‐alone* trials on which the subject was attending to (or near) temporal position 1, from those trials on which they were attending to position 2, or any other position, and calculate FA rates precisely associated with that temporal position, then under this scenario, all FAs would have the same expected value, and therefore, random assignment of no‐signal trials to different temporal positions would be entirely valid. (2) There *is* a modulatory effect of the entraining stimulus that results in subsequent modulation of the variance of internal noise that limits signal detection in a manner that would yield modulatory FA rates as a function of the temporal position to which the subject (unknown to the experimenter) was attending. What then is the effect of random assignment of no‐signal trials to different temporal positions on *d′* measurements given a true modulatory pattern of internal noise? We show below that such random assignment serves only to diminish any M‐shaped pattern of *d′* curves; that is, it works *against* our hypothesis and in favour of the null.

**FIGURE 4:**
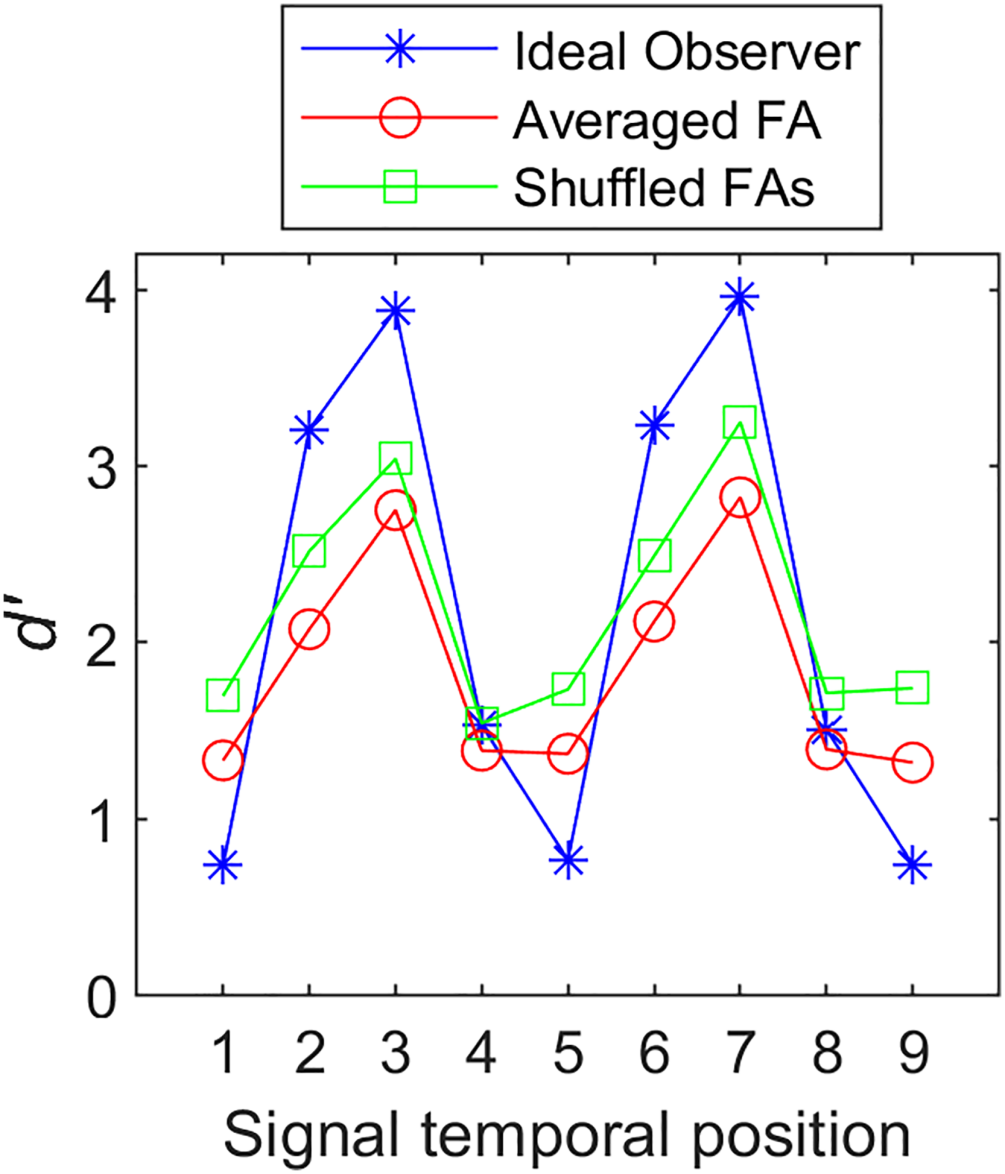
*d*′s determined from Monte Carlo simulations using different approaches to calculating FA rates: (1) ideal observer, (2) averaged FA rates and (3) shuffled FA rates

We conducted ideal‐observer analysis for the signal‐known‐exactly (SKE) model in which the observer has complete information about the statistics of the signal and noise (Green & Swets, 1966; Hautus et al., 2021). To maximize performance, the observer attends to specific (and correct) temporal positions during the signal trials but also to that position during the noise‐alone trials (true for all nine positions). This, by definition, allows accurate estimates of hit and FA rates for each of the nine temporal positions. Figure 4 shows results of Monte Carlo simulations based on 10,000 runs under each of three conditions: (1) randomly assigned ‘no‐signal’ trials to each temporal position in calculating FA rates (i.e., shuffled FAs), (2) *averaging* results for all no‐signal trials to calculate one grand FA rate applied to all temporal positions in calculating *d′*s and (3) ideal assignment of trials assuming that there is in fact a modulatory effect of internal noise on FA rates.

We found that the only effect of random assignment of no‐signal trials to different temporal position is to diminish the M‐shaped pattern of *d′*. In other words, random assignment works in favour of the null hypothesis and only serves to reduce the likelihood of obtaining a bicyclic pattern, and for this reason, its usage to confirm bicyclic effects is valid from a statistical inference standpoint. 6 The fact that even after random assignment of ‘no‐signal’ trials we still observe M‐shaped patterns supports the conclusion that signal detection follows bicyclic modulation. It is noteworthy that random assignment of no‐signal trials in estimating FA rates and averaging results from all no‐signal trials generate similar results, though the former produces slightly higher *d′*s, possibly because averaging no‐signal trials introduces correlation across temporal positions whose differences we are trying to estimate. We should add here that in calculating *d′*s for individual subjects, Sun et al. used the exact same procedure as we have in estimating FA rates by assigning ‘no‐signal’ trials to different temporal positions based on their own trial‐by‐trial labelled data (see their fig. S5 and our footnote 2). In fact, our analysis perfectly reproduced their 23‐panel *d′* curves. Surprisingly, Sun et al. only published the individual‐subject *d′* curves (23 panels in their fig. S5) but not the averaged *d′* curve (as they had for hit rates) which would have produced the same bicyclic curve shown in our Figure 3a.

**FIGURE 5:**
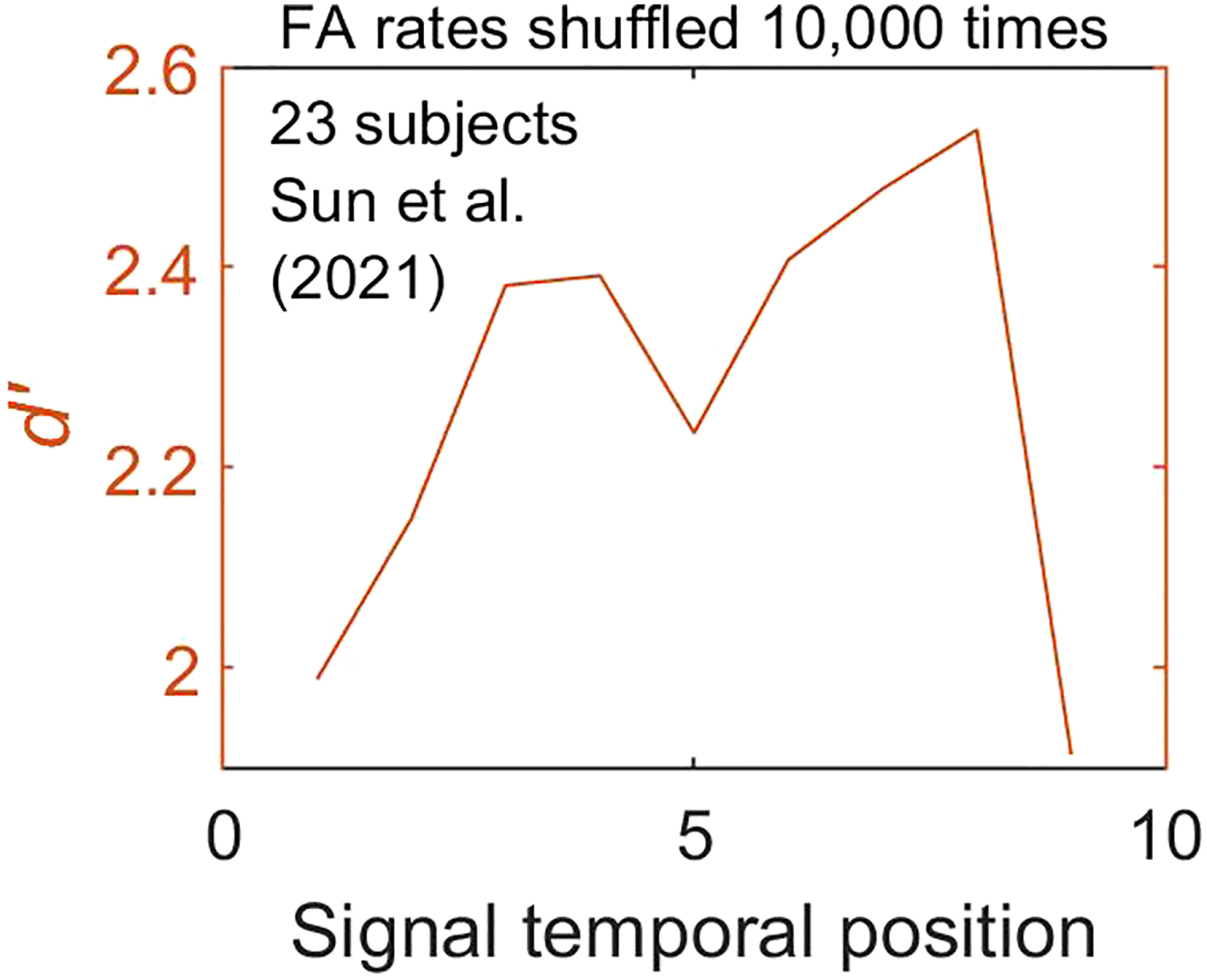
Result of shuffling FA rates across temporal position separately for each of 23 subjects. Average of 10,000 such shuffles

To confirm that the observed bicyclic pattern in Sun et al.'s data is not the result of a particular random assignment of ‘no‐signal’ trials to different temporal positions, we conducted simulations in which the FA rate for each subject at each temporal position was randomly assigned to a different temporal position for that subject, resulting in perturbation of FA rates across the nine positions. The mean *d′* curve (all 23 subjects) for the optimum SNR condition calculated from the average of 10,000 such reshuffling of FA rates is shown in Figure 5. Results suggest that the bicyclic pattern is robust and not a consequence of an aberrant pattern of random assignment of no‐signal trials. 7

### 3.2 Inferences from psychometric functions

**FIGURE 6:**
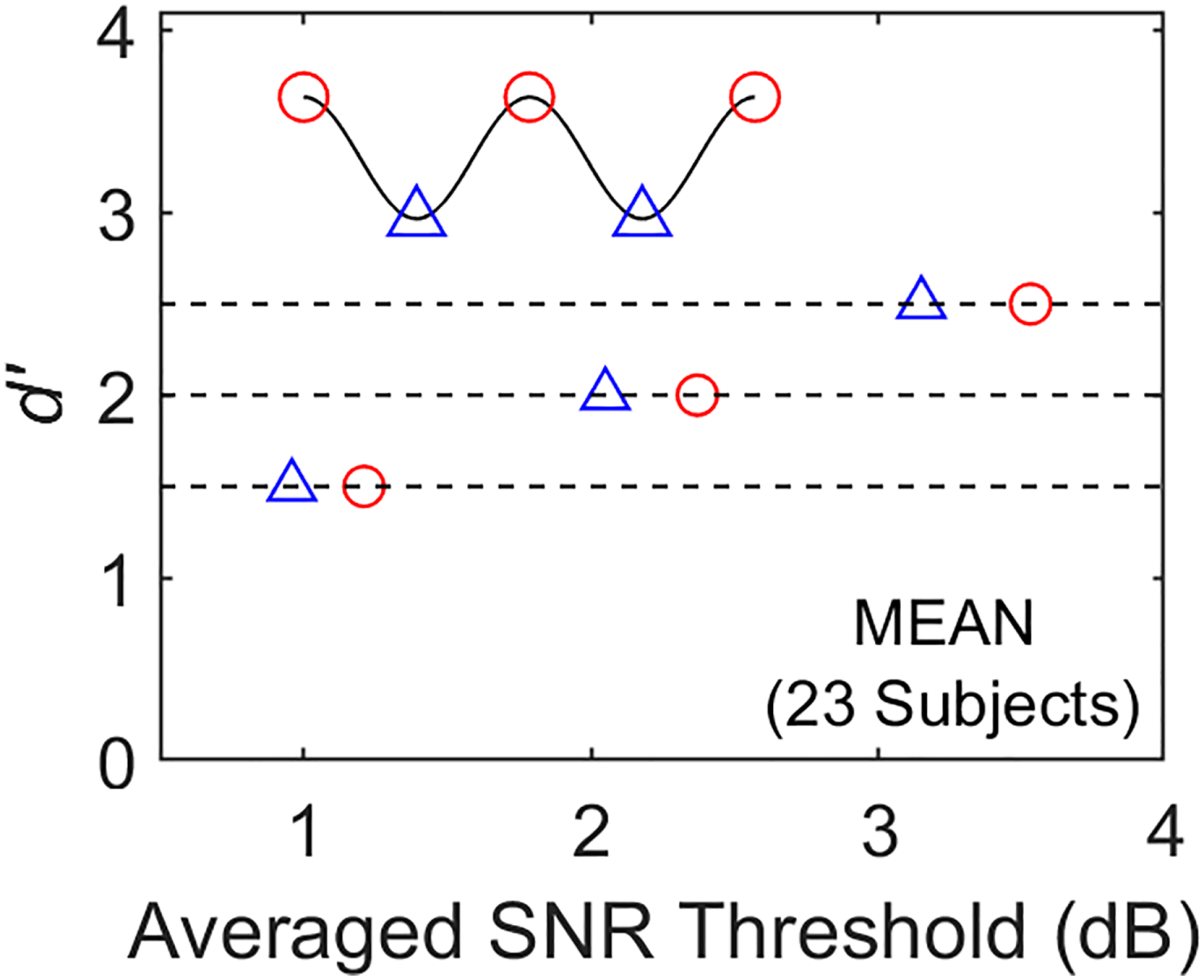
SNR thresholds at 3 *d*′ levels calculated from psychometric functions averaged across 23 subjects from Sun et al. (2021) for each of two ‘phase categories’ (peak‐phase = red; trough = blue). Note that the peak‐phase category requires a higher SNR threshold to reach a performance level (*d*′) equivalent to that for the trough category (envelope minima). Legend is shown in upper left (sinusoid).

We conducted additional analyses on the raw data of Sun et al. that made use of a larger portion of their data set. Specifically, we measured performance at expected peak‐phase compared to trough (envelope minima) target positions based not just on a single SNR but on 3‐point psychometric functions that made *simultaneous* use of three SNRs, tripling the number of trials from which thresholds are estimated. First, for each subject, we measured *d′* psychometric functions for three peak‐phase and two trough‐phase temporal positions (see sinusoidal legend in Figure 6). We then averaged these functions across the 23 subjects as well as within a ‘phase category’ (peak or trough). This resulted in two 3‐point psychometric functions, one for each phase category. We defined SNR threshold to be the point at which regression fits to each psychometric function crossed a specific performance level (e.g., *d′* = 1, 2 or 3). Results shown in Figure 6 demonstrate that when performance is measured in *d′* units using a larger data set comprising three SNRs, temporal positions at the expected dips of the modulation envelope consistently generate better performance than those at the peaks for any of the three *d′* levels at which performance was estimated (i.e., a higher SNR value is needed to reach an equivalent *d′* performance level).

Sun et al. also performed a supplemental analysis of hit‐rate curves in which they excluded subjects with high FA rates. The goal was to eliminate ‘biased subjects’. This, however, misinterprets the meaning of bias in signal‐detection terms. Bias here does not mean high FAs. A subject could be biased *against* responding ‘yes’ and generate very low FA rates. Evaluating performance based on hit rates is biased because it only examines one aspect of performance (hits) and excludes the complementary and inherently linked FA rates. One without the other is a meaningless measure. In a single‐interval yes–no task, the only truly unbiased case occurs when the subject places their decision criterion at precisely the intersection of the *internal* ‘noise‐alone’ and ‘signal‐plus‐noise’ probability distributions where the likelihood ratio is equal to unity (Green & Swets, 1966; Hautus et al., 2021). This is of course unknown to the experimenter and inconsistent across subjects, so the only way to ensure unbiased measurements is to combine hit and FA rates in estimating the detection index *d′*. Sun et al. further justify their use of hit rates as their main approach by noting ‘*One might argue that when participants' overall hit rate is too high (clearly above threshold) or too low (clearly below threshold), it reduces the likelihood of observing fluctuations across different temporal positions … If there was any truth in this conjecture, one would expect to observe a clearer presence of the entrainment effect across participants whose overall hit rate is confined within a narrower range, … we computed a range‐of‐interest, … then selected … participants whose overall hit rate … was enclosed in this range … Results from this more restricted analysis showed that the average modulation strength of selected data is not significantly above zero*’. The flaw in this logic can easily be demonstrated by comparing the unbiased performance of subjects who have similar hit rates. Subjects 222 and 223 have identical hit rates of 0.55556 but very different *d′*s (2.68 vs. 1.97) with subject 222 significantly outperforming subject 223 in detecting the signal. Therefore, it is not methodologically valid to attempt to equate subject performance based on hit rates or to define a range‐of‐interest hit rate in order to eliminate ‘biased’ subjects or outliers with markedly different performance levels.

## 4 ERRORS IN CALCULATING ENTRAINMENT STRENGTH

A second major flaw in Sun et al.'s analysis has to do with the way in which they define the magnitude of entrainment, what they refer to as ‘modulation strength’, on which they base nearly all of their analysis and from which they draw nearly all of their conclusions. This measure, which is the normalized Fourier transform of the performance curves (mostly hit‐rate curves), is not only erroneously calculated but is also unreliable in estimating the strength of entrainment. In defining ‘modulation strength’, Sun et al. note that they ‘*performed Fourier transforms of the curves of target detectability (hit rates) for each participant. Given that each curve only contained 9 temporal locations covering a duration of 667 ms, a Fourier transform of these data only provided accurate power estimation at 4 frequencies, corresponding to 1.33 Hz, 2.67 Hz, 4 Hz, and 5.33 Hz. Among these four frequencies, we selected 2.67 Hz—which is closest to the modulation rate (3 Hz) in the experiment—to be the frequency of interest and measured its power for each participant*’. In over 30 places in their paper they note the use of 2.67 Hz as their base analysis. Their Fourier transform calculations, however, are in error. In Fourier analysis, the frequency component spacing is derived from the inverse of the duration of the function whose spectrum is to be calculated. There are 9 points at which performance is measured, temporally spaced at a quarter of a cycle of the 3 Hz sinusoid with successive points separated by 83.33 ms. The ‘duration’ of the performance curve is equal to exactly 2 cycles at 3 Hz, or 666.66 … ms. The frequency component spacing is therefore 1/0.666 … or exactly 1.5 Hz. Thus, the fast Fourier transform (FFT) contains energy precisely at 1.5, 3 and 4.5 Hz (not 1.33, 2.67, 4 and 5.33 Hz). In fact, the discrete Fourier transform of this function has no energy at 2.67 Hz to be measured. Because the sampling *rate* of the performance curves is 12 Hz (4 points per cycle at 3 full cycles per second), the frequencies at which spectral energy can be reliably measured have to be below the Nyquist limit to avoid aliasing (i.e., half the sampling rate), and therefore, measurements are restricted to component frequencies below 6 Hz (1.5, 3 and 4.5 Hz). We trace the error made by Sun et al. to miscalculating the performance curve ‘duration’. They appear to have assumed that 9 points result in 9 quarter‐cycle segments (instead of *n* − 1 segments). Each segment is 83.33 ms in duration, hence 9 × 83.33 = 750 ms, the inverse of which is 1/0.75 or 1.33 Hz component spacing which results in the erroneous estimates of 1.33, 2.67, 4 and 5.33 Hz. 8 This mathematical error has important implications. For example, Sun et al. report data and statistical analyses on modulation at 5.33 Hz and draw inferences as to presence of harmonic energy in a subset of their subjects and absence of harmonic effects in modulation in most subjects. This analysis is entirely invalid as the correct rate of modulation on which they have based their analysis is 6 Hz, which falls precisely on the Nyquist (folding) frequency, and hence, the waveform for which they have measured modulation strength cannot be unambiguously reconstructed at the frequency of interest due to signal aliasing.

**FIGURE 7:**
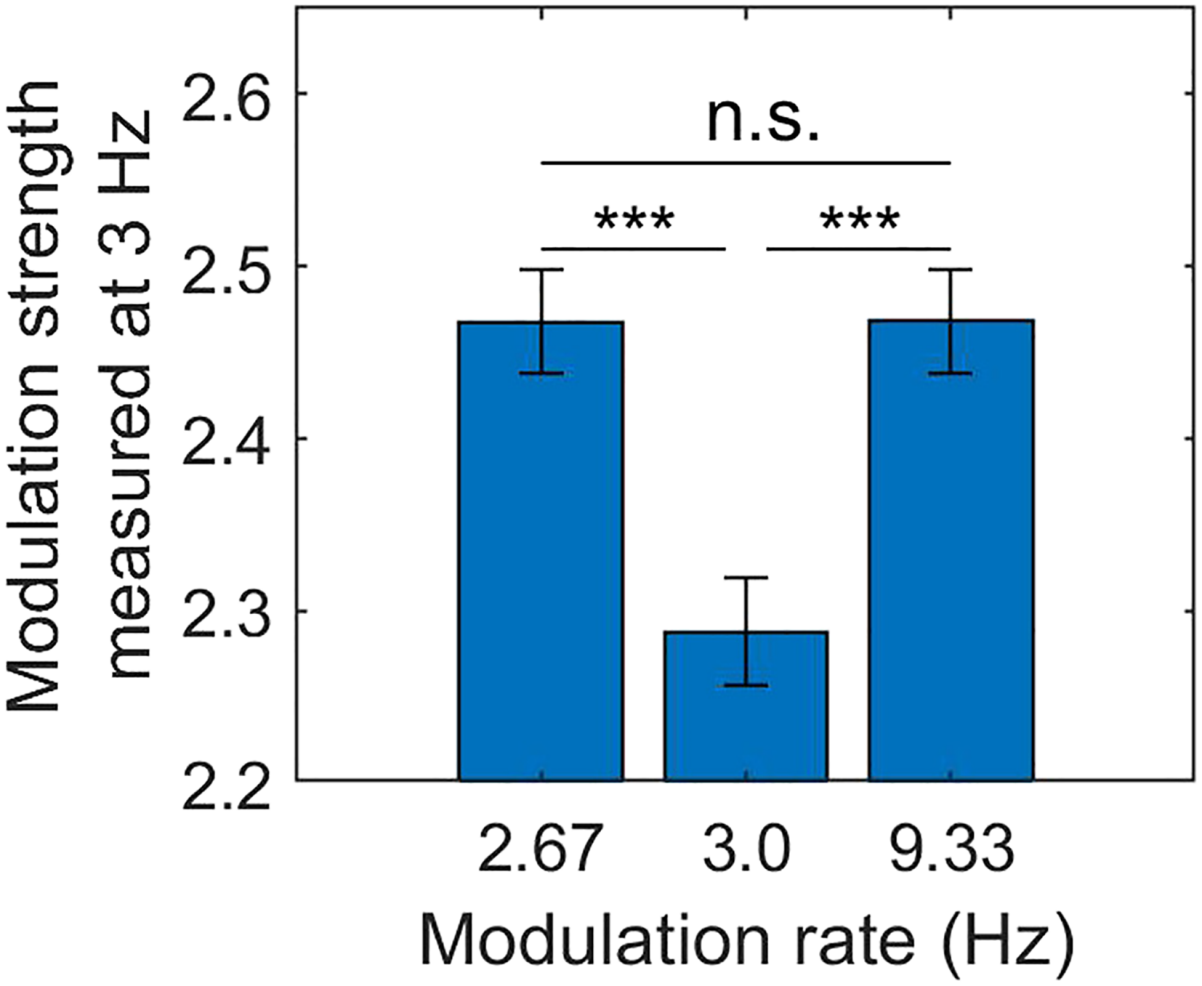
Potential flaws in using ‘modulation strength’ as a measure of entrainment. The method is shown here to generate weaker estimates at 3 Hz in response to a 3 Hz sinusoid than at 3 Hz in response to off‐frequency sinusoids (2.67 and 9.33). Error bars represent ±1 standard deviation (see text and Figure S3 for details).

Separate from this mathematical error, there is a more fundamental concern with using the Fourier transform to estimate ‘modulation strength’, one that Farahbod et al. (2020) had previously cautioned about. When using very brief signals, such as the 2 cycle waveform associated with behavioural performance curves, the duration of the waveform itself (i.e., the analysis window), regardless of the shape of the waveform within that window, will enhance energy at some regions of the spectrum and diminish energy at others. This can confound ‘modulation strength’ measurements. Theoretically, a 2 cycle sinusoid is generated by multiplying an infinitely long sine wave with a rectangular (boxcar) window in the time domain. The spectrum of such a waveform is the convolution of the spectra of the infinitely long sinusoid (a vertical line with zero bandwidth) with that of the rectangular window (the well‐known sinc function). The sinc function has zero‐crossings in the magnitude spectrum at the inverse of the window's duration (Rabiner & Gold, 1975). The sinusoid's frequency and the width of the temporal window constraining that sinusoid jointly determine the position of these zero‐crossings in the spectrum. The effects of the sinc function on the spectrum can constructively or destructively alter the amplitude of frequency components used to measure ‘modulation strength’ (see Figure S2). Furthermore, for very brief sinusoidal pulses, the starting phase itself affects the spectral profile of that waveform, and because the duration of the analysis window is a subharmonic of the modulation rate under study (a duration equal to exactly 2 cycles of modulation), by definition, spectral energy will be generated at the expected modulation rate. 9 These interactions may potentially produce false positives or misses in identifying whether energy at a given frequency component results from entrainment or from signal‐processing artifacts. For example, in our paradigm (and that of Sun et al.), a frequency of 2.67 Hz (the value that Sun et al. erroneously calculated) or 9.3 Hz (which is above the Nyquist limit) will generate ‘modulation strength’ measurements at 3 Hz that are actually *stronger* than that generated by a true 3 Hz sinusoid, making such measurements potentially unreliable in estimating entrainment (Figures 7 and S3). We therefore caution against use of the discrete Fourier transform in determining the strength of entrainment when analysing very brief waveforms because their spectra are too severely altered by the spectral profile of the rectangular window that defines their duration.

What then is, in our opinion, a more appropriate method for detecting forward entrainment in performance curves? We have previously considered a number of other measurement methods (e.g., FFT, autocorrelation and cross‐correlation with a single cycle sinusoid as discussed in Farahbod et al., 2020), and others have suggested a number of alternative methods based on parametric regression‐based methods (Zoefel et al., 2019). We have come to the conclusion that the use of parametric statistical models to determine overall effects coupled with hypothesis‐driven post hoc tests of antiphasic (or other) effects is our preferred approach to detection of psychophysical entrainment, at least when considering the bicyclic antiphasic functions that we have observed in several studies.

## 5 ADDITIONAL CONSIDERATIONS

There are a number of other observations about the findings of Sun et al. that are worth considering here. Sun et al. note that the methodological differences between their two experiments (‘conceptual’ and ‘exact’ replication) are relatively minor and inconsequential. If this is the case, then one has to explain several significant differences in the results of these experiments. First, as they acknowledge, the two experiments produced peak performance at different temporal positions (time positions 5 and 6 in experiment 1 and positions 6 to 8 in experiment 2). Second, the average starting phase of forward entrainment, for the ~35% of subjects who showed entrainment, was significantly different across the two experiments. In their experiment 1, subjects had averaged starting phases of 102° (0.6π rad), whereas those in their experiment 2 had an average starting phase of 220° (1.22π rad). Interestingly, the latter is less than a quarter of a cycle (0.14 cycles) away from the antiphasic position (270°, 1.5π) and approximately a 10th of a cycle away from that reported by Hickok et al. (262°, 1.46π). Note that the duration of the tonal signal (50 ms) is equivalent to 0.15 cycles of modulation at 3 Hz, whereas the average difference in starting phases of performance curves between Hickok et al. (2015) and Sun et al. (2021) is 42°, or 0.12 cycles at 3 Hz, less than the range of phases covered by the duration span of the 50 ms tone.

As mentioned above, a notable difference between the findings of Hickok et al. (2015) and Sun et al. (2021) is the significantly larger intersubject variability in the latter study. Sun et al. contend that ‘*larger cross‐participant variability is generally expected with increased sample size’*. Increasing subject sample size improves the precision with which the population variance in performance is estimated but has no systematic effect on the comparative size of the variances of two samples if they are unbiased estimators of the population variance. These sample variances, however, will be different if the nature of the underlying populations from which they are sampled are different. One potential population difference is subject experience. Subjects in Hickok et al. (2015) were highly experienced having previously participated in a number of auditory psychophysical tasks (graduate students and postdocs). Sun et al. do not report whether their participants had extensive experience or were experimentally naïve (they used 47 subjects who completed one or two experimental sessions). While we do not know the level of prior psychophysical experience of their subject population, this may be a factor worth considering, especially given new data from our lab that demonstrates more variable patterns of performance for inexperienced and perhaps less motivated subjects (Saberi & Hickok, 2021).

An interesting finding reported by Sun et al. (2021) is that ‘*a subset of participants (~36%) exhibited the entrainment effect in behavioral performance*’ even when using hit rates as a performance measure. Bauer et al. (2015) similarly reported that 40 of their 140 subjects (28%) showed forward entrainment in a pitch‐discrimination task. As noted earlier, if Sun et al.'s data are reanalysed using *d′*, a bicyclic pattern with an antiphasic dip is obtained at the full population level. Nonetheless, even by their own measure of performance (hit rates), the finding that a segment of the population does show the effect and a segment does not is actually confirmation of the existence of the effect in a subcategory of subjects and to conclude that an absence of statistical significance at the full sample population level is evidence against existence of forward entrainment is misleading. There are several auditory phenomena that are consistently observed in a segment of normal‐hearing populations and not in others for reasons that may range from experience to genetics (Assaneo et al., 2019; Chubb et al., 2013; Ho & Chubb, 2020; Mednicoff et al., 2018).

As to where precisely should one expect to observe the peaks and dips of performance curves in forward entrainment, while the pattern of entrained activity for *simultaneous* entrainment (in which the entraining and entrained processes are concurrently active) may follow a precise periodicity that mirrors that of the entraining oscillator due to iterative phase resetting, in forward entrainment, the oscillatory patterns will not necessarily (and likely will not) have the periodicity precision of the entraining oscillator. Performance curves in forward entrainment are often not sinusoidal, they have sharp peaks, the second peak is usually larger than the first, the antiphasic dip is not as low as those associated with the first and last temporal positions and the curves appear to be phase modulated in which a drift of the second cycle to a lower frequency may be observed (Farahbod et al., 2020; Hickok et al., 2015; Saberi & Hickok, 2021). Evidence of a phase drift is also present in the visual entrainment data of de Graaf et al. (2013; see their fig. 3). Therefore, what one can say with certainty is that there is a modulatory effect in performance after termination of the entraining stimulus, but that the precise position of peaks and dips (although consistent across listeners) must be empirically measured given that there will likely (and understandably) be a phase drift when the driving modulator is removed and the system gradually reverts to its default mode.

## 6 CONCLUSIONS

Finally, we would also like to briefly comment on Sun et al.'s speculation as to why there were contrasting findings between their study and Hickok et al. (2015). They attribute this to a number of potential factors, including the details of the experimental environment, an accidental underrepresentation of listeners who do not exhibit forward entrainment (note that even they report that 36% of their subjects show entrainment) or subject‐dependent entrainment phases which would flatten out bicyclic effects at the group level. They dismiss this latter explanation, arguing that the previously reported subject‐specific phase dependency (Henry & Obleser, 2012) was observed for FM sounds, whereas the maskers used in Hickok et al. and Sun et al. were amplitude‐modulated sounds (AMs). However, there is significant evidence for FM‐to‐AM conversion in the auditory periphery as the instantaneous frequency of an FM signal sweeps through the passband of auditory filters (Henning, 1980; Hsieh et al., 2010; Hsieh & Saberi, 2010; Saberi, 1998; Saberi & Hafter, 1995). This conversion pattern is complex and dependent on both the integration time constant of the system and the relative position of an auditory filter to that of the FM carrier. The induced‐AM signal resulting from a sinusoidal FM will have a rate that is twice that of the FM at the output of a filter centred on the FM carrier and match that of the FM rate if the sweep only passes through the lower (or upper) skirt of an off‐centre filter (but not both). Off‐frequency listening away from the FM's carrier frequency will therefore result in use of AM cues equivalent to the entraining FM rate. Furthermore, several subjects in the study by Henry and Obleser (2012) show a clear antiphasic pattern of behavioural performance relative to the entraining stimulus phase (their figs 3 and S2), whereas others show an in‐phase pattern (or at a different phase). This may be partially related to implicit subject‐specific strategies in off‐frequency listening at the output of a filter that is either above or below the FM's carrier frequency. Listening at the output of a filter above the FM carrier when the instantaneous frequency of the FM is at its lowest value (antiphasic) will maximize SNR because the induced AM at the output of that filter will be at its minimum amplitude. Off‐frequency listening at the output of a filter below the FM carrier will generate the opposite pattern. Thus, there are significant shared mechanisms in how the auditory system processes FM and AM sounds and the subject‐specific phase dependency in entrainment reported for FM sounds could have relevance to AM signal processing.

Sun et al. further speculate on other potential factors that may result in an absence of forward entrainment. They suggest, citing Bauer et al. (2015), that when there are conflicting spectrotemporal cues in the entrainment process (as when the entraining stimulus and the signal are not of the same stimulus class), one may fail to observe forward entrainment. Sun et al. then note that ‘there is no direct correspondence between the spectral content of the entraining signal (broadband noise) and target stimulus (1 kHz tone)’ in their design or that of Hickok et al. (2015). They conclude that it is therefore unclear why an AM *noise* would trigger entrainment that would facilitate the detection of a *tone* pulse (i.e., different classes of sounds). Setting aside the fact that the noise spectrum contains energy at the frequency of the tone that it masks, what Sun et al. fail to recognize is that the entraining AM noise, as suggested by Simon and Wallace (2017), entrains *against* the noise that limits signal detection. From this standpoint, the similarity in spectral characteristics of the entrained and entraining noise can facilitate better isolation of the target to be detected during expected dips. Importantly, however, our reanalysis of Sun et al.'s data using unbiased dependent measures argues that appeal to such explanations is generally unnecessary as their study demonstrates forward entrainment when appropriate dependent measures are used.

## AUTHOR CONTRIBUTIONS

K. Saberi and G. Hickok wrote the manuscript and performed the data analysis.

## CONFLICTS OF INTEREST

The authors declare no conflicts of interest.

### PEER REVIEW

The peer review history for this article is available at https://publons.com/publon/10.1111/ejn.15816.

## Supporting information


**Figure S1.** Same data as shown in with error bars representing one standard error of the mean.
**Figure S2.** A) Time domain representations of two rectangular windows of differing durations (500 and 666.6 ms). B) The spectrum of each is the sinc function with zero‐crossings at the inverse of each window's duration. C) The spectrum of a sinusoidal pulse with an arbitrary frequency of 7 Hz shown to demonstrate how false positives can be generated in overestimating entrainment at 3 Hz, or how entrainment strength can be underestimated if the enhanced energy is antiphasic to the frequency of interest. D) For a fixed duration of 666.6 ms, which is equal to 2 cycles of a 3 Hz sinusoid, energy at a number of frequencies (both below and above 3 Hz) can enhance or diminish activity at 3 Hz, resulting in false positives or misses when using “modulation strength” as defined by Sun et al. (2021) to quantify entrainment. E & F) Zero‐padding a rectangular window does not affect the shape of the sinc function or the positions of peaks and zero‐crossings, but rather only increases the frequency resolution with which the function can be plotted.
**Figure S3.** This figure shows how “modulation strength”, the measure developed by Sun et al. to quantify entrainment, produces unreliable results when analyzing brief (impulsive) waveforms. A) Waveforms generated at the 9 temporal positions that define the behavioral curves using one of 3 underlying sinusoidal frequencies (2.67, 3.0, and 9.33 Hz). Legend is shown in the bottom panel. Here we refer to the 3‐Hz waveform as the target frequency and the other two frequencies as “off‐frequency”. The ideal waveform that generates the antiphasic M‐shaped bicyclic pattern is a 3‐Hz sinusoid (negative cosine phase). This should produce the strongest “modulation strength” measured at 3 Hz. B) The functions for 2.67 and 9.33 Hz produce overlapping points because of signal aliasing. C) Spectra of sinusoids at 2.67, 3.0, and 9.33 Hz based on the 9‐point waveforms shown in panel A. The two off‐frequency signals produce a sharper spectral peak at 3 Hz than the 3‐Hz sinusoid. This is partly due to the large dc (direct current) component, resulting in spectral asymmetry associated with the 3‐Hz waveform. Intuitively, this can be observed in panel A (green trace) where the 3‐Hz waveform has two maxima and three minima, producing a non‐zero mean amplitude. For the off‐frequency waveforms the dc components are closer to zero, and hence better symmetry about the 3‐Hz peak. Consequently, “modulation strength” measured for the off‐frequency waveforms are stronger at 3 Hz than that measured for an actual 3‐Hz target sinusoid (see Figure 7). “Modulation strength” means and variances in Figure 7 were obtained from 5000 runs of 1000 permutations each of temporal positions of amplitudes (panel A in the current figure) in obtaining a baseline measure for comparison to energy at 3 Hz as described by Sun et al. (2021).

## Data Availability

matlab programs and data used to generate figures in this paper are available at UCI's Data Repository (DRYAD): https://doi.org/10.7280/D1PQ5N.

## Footnotes

^1^
Some of the methodological differences between Hickok et al. (2015) and Sun et al. (2021) include the following: Sun et al. used a staircase procedure to set signal levels individually for each subject (experiment 1), used practice runs prior to the actual experiments that exposed subjects to high SNR levels, participants were restricted to a response time window of 1.5 s, intertrial interval was randomized (between 1 and 2 s) and a pseudorandomized procedure was used in assignment of trial types instead of a fully randomized design used by Hickok et al. (2015). In their pseudorandomized design, they forced an equal number of trials for signal and no‐signal conditions (as well as an equal number of trials per temporal position and per signal level). Psychophysicists typically avoid use of pseudorandom designs largely because they introduce serial dependencies near the end of runs that can affect subject response strategy. An examination of the raw data of Sun et al. (2021), which they have made publicly available (https://doi.org/10.17617/3.5c), shows that such non‐independence resulted in aggregation of specific trial types, for example, ‘signal‐present’ trials, at the end of many run, for example, subject 205, run #18; subject 207, run #13; and subject 213, run #7. This serial dependency can, for example, increase the likelihood of FAs on runs in which the prior expectancy has changed in favour of responding ‘yes’ near the end of a run (i.e., a decision criterion shift). Subjects can pick up on these trends and develop effective response strategies (same argument applies to signal level and temporal positions). The authors state that balancing of trial types was ‘particularly crucial to prevent participants from developing response biases’. We argue that in fact such pseudorandomization almost ensures development of response biases within a run and especially near the end of a run.

^2^
In the following analysis, we focus exclusively on their experiment 2 (‘exact’ replication). By convention, we assumed a small inattention rate in setting a ceiling hit rate of 0.99 and a floor FA rate of 0.01 (Green, 1995; Hautus et al., 2021; Saberi & Green, 1997). This avoids the practical problem of *d′* = ∞ when estimating performance in small samples. For each combination of temporal position and signal level, FA was determined from the raw data of Sun et al., which for *each* trial included a designation of signal temporal position and level, as well as whether or not a signal was presented on that trial (i.e., the same procedure used by Sun et al. to generate their fig. S5). This allowed independent estimates of FAs at each temporal position. In their original manuscript, which Sun et al. graciously shared with us, only hit‐rate measurements were made. The peer review history of their paper, made publically available by the European Journal of Neuroscience (https://publons.com/publon/10.1111/ejn.15367), shows that the reviewers cautioned against this approach. In spite of these cautions, the authors maintained hit rates as the basis of their primary analysis, largely dismissing use of FAs in calculating *d′* as just adding noise to the data. In the final version of their paper, the authors, to their credit, do show cursory measurements using *d′*s, but this analysis is incomplete, failing to take the extra step of reporting the averaged *d′* curve that shows a bicyclic pattern (our Figure 3a) and failing to conduct the necessary statistical analyses that would have confirmed an antiphasic dip in performance (see also our critique of their ‘modulation strength’ measurements).

^3^
For clarity of presentation, error bars have been excluded from this figure. However, these error bars are shown in Figure S1 as *d′* and proportion‐correct data plotted in separate panels.

^4^
Similar results were found for statistical analysis of proportion‐correct data. The omitted subject (subject 204) generated an outlier pattern of performance with an antiphasic dip that occurred one time point earlier at temporal position 4 (i.e., phase advanced by a quarter of the modulation phase). This phase drift created a counter‐modulatory effect when compared to the group‐level pattern based on the 22 other subjects.

^5^
The mean optimum SNR level was 2.17, which is close to the original SNR level of 2.

^6^
We considered a number of additional modulatory patterns of internal noise for no‐signal trials, including patterns derived from Sun et al.'s hit‐rate data (because observers would not have a priori knowledge whether or not a signal will occur on a given trial), a flat (unmodulated) pattern and a counter‐modulatory pattern. In all cases, M‐shaped patterns diminished relative to that of the ideal observer. It is of interest to note that the slight rightward slant to the M‐shaped functions generated by the ideal observer (Figure 4) is also seen in Sun et al.'s data (Figure 3a,b). This may be related to the fact that the 50 ms tone is slightly off‐centre (by 25 ms) relative to the expected modulation phase. The offset occurs because the tone's *onset* (not its centre) is aligned with each temporal position (i.e., *onset* of the first tone, not its centre, is positioned at temporal position 1 where the entraining modulation ends).

^7^
It is also worth considering how entrainment may affect a listener's decision criterion. If true sensitivity remains constant but criterion modulates, random assignment of no‐signal trials could potentially manifest as modulation of detection performance. This, however, is unlikely because the listener's decision criterion, which is often considered to be a more deliberative process that builds over time, would have to shift multiple times (4 to 8 times) within a span of approximately half a second to generate the observed cyclic pattern of performance. Although evidence exists for rapid capture of transient attention (Dugué et al., 2016; Landau & Fries, 2012; VanRullen et al., 2007), no prior study has shown such remarkably rapid shifts in decision processes. Nonetheless, the contributions of rapid shifts in criterion can be empirically estimated in future studies by presenting an unequal proportion of ‘signal‐present’ trials at different temporal positions. For example, by assigning a larger number of ‘signal’ trials to positions at which we have observed poorer performance (the expected modulation peaks), the participant's criterion should shift to a higher rate of ‘yes’ responses at those temporal positions and conversely a lower ‘yes’ response rate at positions where high performance had been observed. This should flatten out the previously observed cyclic curve. We would, however, predict no effect of rapid criterion shift on forward entrainment, that is, that the cyclic patterns observed in Figure 3 will likely be preserved.

^8^
The theorem we describe here is that the Fourier transform of a time series is a decomposition of that function into sinusoids that are harmonics of the fundamental frequency of the time series being analysed (Körner, 1989; Rabiner & Gold, 1975). The critical factor here is *duration*, the inverse of which is the waveform's fundamental frequency independent of the number of samples or sampling rate (the latter variables may be used to determine duration). This can be intuitively demonstrated by considering a 1 s waveform whose FFT (or DFT) has component spacing of 1 Hz (1/duration). At a sampling rate of 12 Hz (used in the current analysis), sample points occur at the start of the waveform (*t* = 0) and at ¼ period spacing of a 3 Hz modulator thereafter. The approach employed by Sun et al. would misidentify the true duration of the waveform by appending a 13th point (within the 1 s frame) to the time series in calculating a frequency resolution of 12/13 or 0.92 Hz. This is because the method double counts the final point as both the last point of the 1 s segment and the first point of the second 1 s segment (had the waveform continued). Consequently, the component spacing of 1.33 Hz estimated by Sun et al. for 2 cycles of a 3 Hz sinusoid erroneously leads to an expected waveform duration of 750 ms. This clearly is false because the exact duration is 2/3 (2 cycles of a 3 Hz modulator) generating the correct spacing of 3/2 or 1.5 Hz (1/duration). For long‐duration waveforms (typical in auditory research), a single sample‐point error out of tens of thousands is insignificant, but for very brief temporal windows with only a few points, one sample point is consequential and precision is critical. [Correction added on 29 September 2022, after first online publication: Footnote was corrected in this version.]

^9^
We encountered this issue initially in Farahbod et al. (2020) where forward entrainment was investigated as a function of modulation rates from 2 to 32 Hz. Because in all cases the duration of the analysis window was 2 cycles of the modulating rate under study (i.e., a subharmonic of that rate), the length of the analysis window was a scalar function of the modulation rate. This yielded spectral energy at 2 Hz when analysing entrainment to 2 Hz modulating noise and 32 Hz when analysing entrainment to 32 Hz modulation. For this reason, Farahbod et al. (2020) cautioned against use of FFTs as a method for quantifying entrainment strength for very brief analysis windows.
