# Supplementary material for: Confirming an antiphasic bicyclic pattern of forward entrainment in signal detection: A reanalysis of Sun et al. (2021)
Source: Eur J Neurosci. 2022 Sep 14;56(8):5274–86. doi: 10.1111/ejn.15816 (PMC9826078; doi:10.1111/ejn.15816)
Supplement: Supplementary file 1 — Figure S1. Same data as shown in with error bars representing one standard error of the mean. Figure S2. A) Time domain representations of two rectangular windows of differing durations (500 and 666.6 ms). B) The spectrum of each is the sinc function with zero‐crossings at the inverse of each window's duration. C) The spectrum of a sinusoidal pulse with an arbitrary frequency of 7 Hz shown to demonstrate how false positives can be generated in overestimating entrainment at 3 Hz, or how entrainment strength can be underestimated if the enhanced energy is antiphasic to the frequency of interest. D) For a fixed duration of 666.6 ms, which is equal to 2 cycles of a 3 Hz sinusoid, energy at a number of frequencies (both below and above 3 Hz) can enhance or diminish activity at 3 Hz, resulting in false positives or misses when using “modulation strength” as defined by Sun et al. (2021) to quantify entrainment. E & F) Zero‐padding a rectangular window does not affect the shape of the sinc function or the positions of peaks and zero‐crossings, but rather only increases the frequency resolution with which the function can be plotted. Figure S3. This figure shows how “modulation strength”, the measure developed by Sun et al. to quantify entrainment, produces unreliable results when analyzing brief (impulsive) waveforms. A) Waveforms generated at the 9 temporal positions that define the behavioral curves using one of 3 underlying sinusoidal frequencies (2.67, 3.0, and 9.33 Hz). Legend is shown in the bottom panel. Here we refer to the 3‐Hz waveform as the target frequency and the other two frequencies as “off‐frequency”. The ideal waveform that generates the antiphasic M‐shaped bicyclic pattern is a 3‐Hz sinusoid (negative cosine phase). This should produce the strongest “modulation strength” measured at 3 Hz. B) The functions for 2.67 and 9.33 Hz produce overlapping points because of signal aliasing. C) Spectra of sinusoids at 2.67, 3.0, and 9.33 Hz based on the 9‐point wavef [file EJN-56-5274-s001.pdf]

Supplemental Figures:
Confirming an antiphasic bicyclic pattern of forward entrainment in signal detection: a reanalysis of Sun et al. (2021)

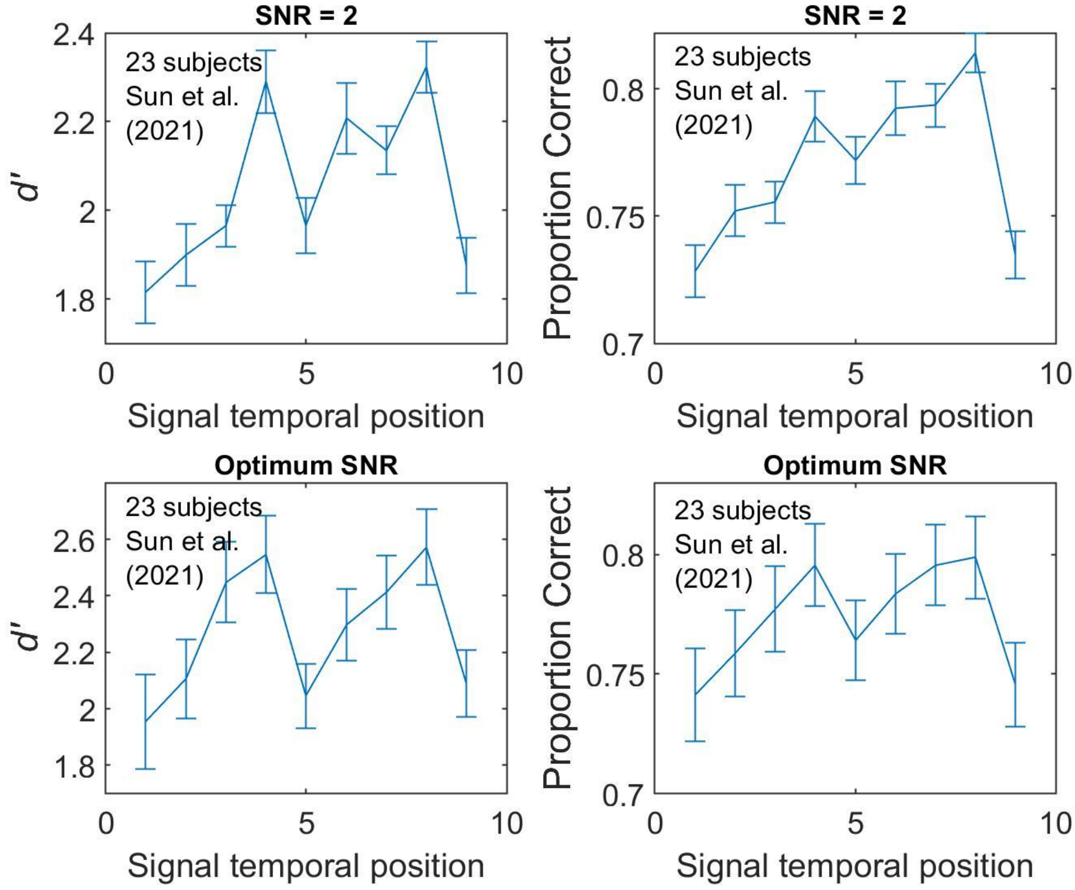

Supplemental Figure 1. Same data as shown in panels A and B of Fig. 3 with error bars representing one standard error of the mean.

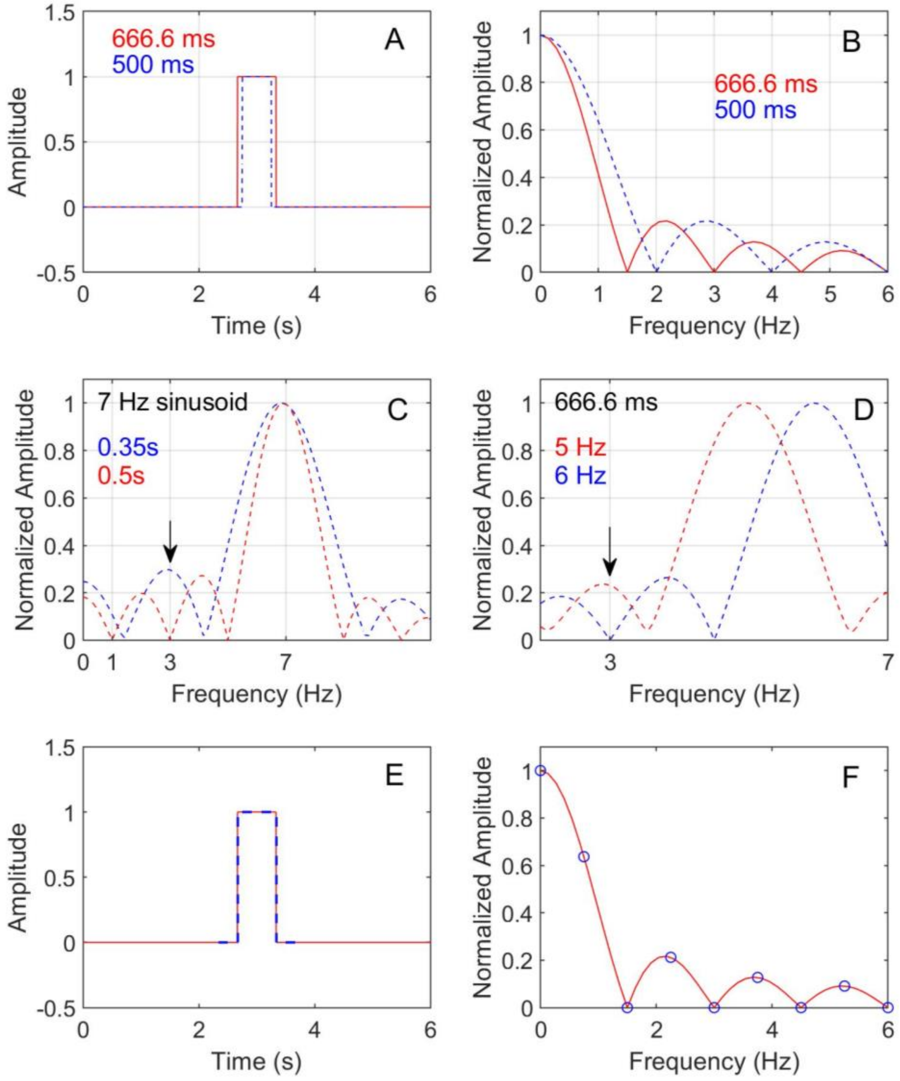

Supplemental Figure 2. A) Time domain representations of two rectangular windows of differing durations (500 and 666.6 ms). B) The spectrum of each is the sinc function with zero-crossings at the inverse of each window's duration. C) The spectrum of a sinusoidal pulse with an arbitrary frequency of 7 Hz shown to demonstrate how false positives can be generated in overestimating entrainment at 3 Hz, or how entrainment strength can be underestimated if the enhanced energy is antiphasic to the frequency of interest. D) For a fixed duration of 666.6 ms, which is equal to 2 cycles of a 3 Hz sinusoid, energy at a number of frequencies (both below and above 3 Hz) can enhance or diminish activity at 3 Hz, resulting in false positives or misses when using "modulation strength" as defined by Sun et al. (2021) to quantify entrainment. E & F) Zero-padding a rectangular window does not affect the shape of the sinc function or the positions of peaks and zero-crossings, but rather only increases the frequency resolution with which the function can be plotted.

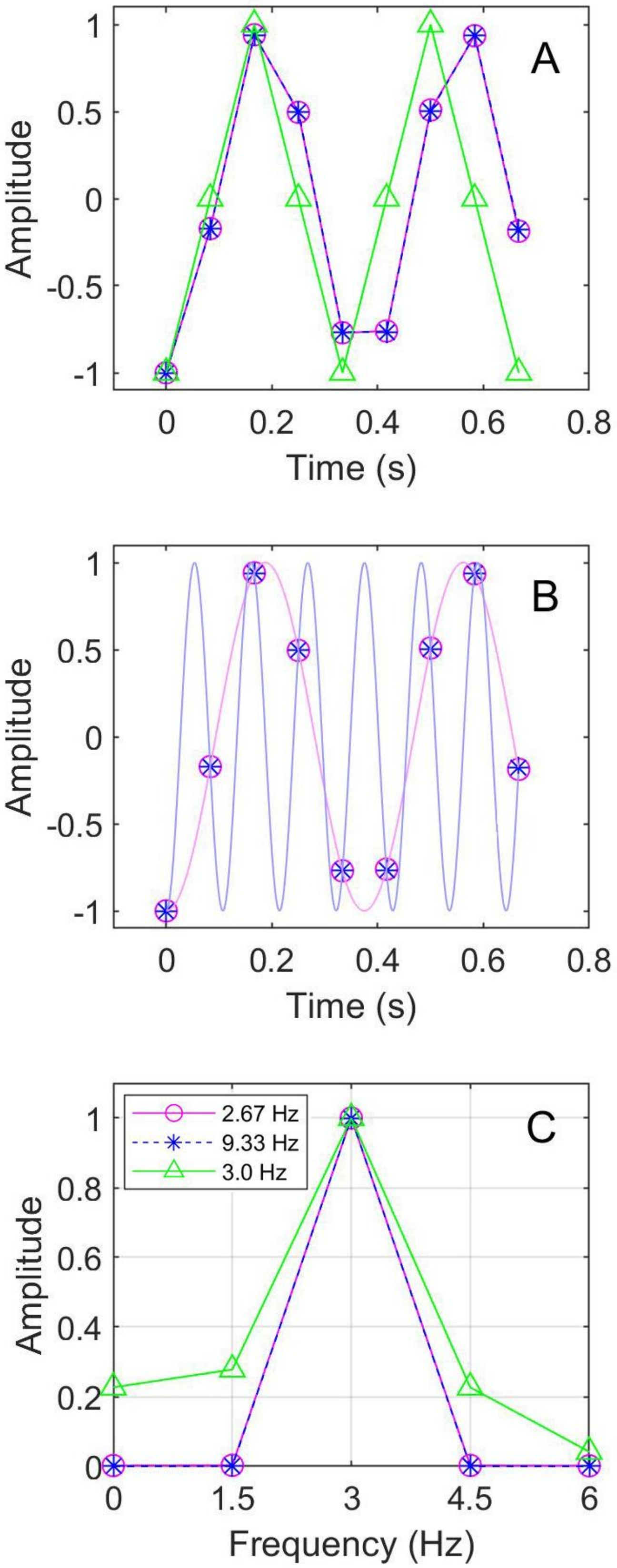

Supplemental Figure 3. This figure shows how "modulation strength", the measure developed by Sun et al. to quantify entrainment, produces unreliable results when analyzing brief (impulsive) waveforms. A) Waveforms generated at the 9 temporal positions that define the behavioral curves using one of 3 underlying sinusoidal frequencies (2.67, 3.0, and 9.33 Hz). Legend is shown in the bottom panel. Here we refer to the 3-Hz waveform as the target frequency and the other two frequencies as "off-frequency". The ideal waveform that generates the antiphasic M-shaped bicyclic pattern is a 3-Hz sinusoid (negative cosine phase). This should produce the strongest "modulation strength" measured at 3 Hz. B) The functions for 2.67 and 9.33 Hz produce overlapping points because of signal aliasing. C) Spectra of sinusoids at 2.67, 3.0, and 9.33 Hz based on the 9-point waveforms shown in panel A. The two off-frequency signals produce a sharper spectral peak at 3 Hz than the 3-Hz sinusoid. This is partly due to the large dc (direct current) component, resulting in spectral asymmetry associated with the 3-Hz waveform. Intuitively, this can be observed in panel A (green trace) where the 3-Hz waveform has two maxima and three minima, producing a non-zero mean amplitude. For the off-frequency waveforms the dc components are closer to zero, and hence better symmetry about the 3-Hz peak. Consequently, "modulation strength" measured for the off-frequency waveforms are stronger at 3 Hz than that measured for an actual 3-Hz target sinusoid (see Fig 7). "Modulation strength" means and variances in Fig. 7 were obtained from 5000 runs of 1000 permutations each of temporal positions of amplitudes (panel A in the current figure) in obtaining a baseline measure for comparison to energy at 3 Hz as described by Sun et al. (2021).
